# New Insights Into the Biogeography of Six *Garra* Species (Teleostei: Cyprinidae) in the Persian Gulf Basin

**DOI:** 10.1002/ece3.73463

**Published:** 2026-04-27

**Authors:** Iraj Hashemzadeh Segherloo, Murtada D. Naser, Amaal G. Yasser, Seyedeh Narjes Tabatabaei, Fardin Shaloei, Shirin Rahmati, Hojat Keshani, Aghil Mansouri, Eric Normandeau, Jörg Freyhof, Eric Hallerman, Erik García‐Machado, Alieh Changizi, Louis Bernatchez

**Affiliations:** ^1^ Department of Fisheries Sciences, Faculty of Natural Resources and Earth Sciences Shahr‐e‐Kord University Shahr‐e‐Kord Iran; ^2^ Institut de Biologie Integrative et des Systemes (IBIS) Universite Laval Québec Canada; ^3^ Museum für Naturkunde, Leibniz Institute for Evolution and Biodiversity Science Berlin Germany; ^4^ School of Environment and Science Griffith University Brisbane Queensland Australia; ^5^ Division of BioInvasions, Global Change and Macroecology, Department of Botany and Biodiversity Research University of Vienna Vienna Austria; ^6^ Plateforme de Bio‐informatique de l'IBIS (Institut de Biologie Intégrative et des Systèmes) Université Laval Québec Québec Canada; ^7^ Department of Fish and Wildlife Conservation Virginia Polytechnic Institute and State University Blacksburg Virginia USA; ^8^ Khuzestan Bureau of Environment Behbahan Khuzestan Iran

**Keywords:** genome, introgression, Last glacial maximum (LGM), mtDNA, river capture

## Abstract

Natural and anthropogenic changes have shaped the geographical distributions of freshwater fishes. During the Last Glacial Maximum (LGM), marine water retreated from the Persian Gulf, and it has been hypothesized that the Tigris River then received all tributary rivers of the present Persian Gulf and reached the Sea of Oman. In this study, we assess the extent and mechanisms of regional movements of the cyprinid freshwater fish *Garra rufa* and its interactions with a few surface‐dwelling and subterranean congeners in the studied region. We analyzed genome‐wide single nucleotide polymorphism (SNP) data and mitochondrial DNA sequences of fishes from the Tigris, Karkheh, Karun, Jarahi, Dalaki, and Mond rivers. It appears that after the LGM, colonization of *G. rufa* into the Mond and Dalaki drainages of the Persian Gulf, unidirectional movements of *G. rufa* from the Dalaki to the western drainages of the Persian Gulf and to the inland Maharlu lake basin may have occurred through river capture. Further, our results, along with published morphological data, indicate no genomic or morphologic differences between *G. rufa* and *G. mondica* as well as *G. gymnothorax*, questioning their status as own evolutionary units. Further, we also found signatures of introgression from *G. rufa* into *G. tashanensis* in the Jarahi drainage.

## Graphical abstract

We present data and inferences supporting the case for post‐LGM secondary contact between eastern and western *G. rufa* populations that became isolated by post‐Pleistocene sea level rise of the Persian Gulf. We suggest river capture as a probable mechanism explaining secondary dispersal of *Garra rufa* populations from eastern to western river drainages.

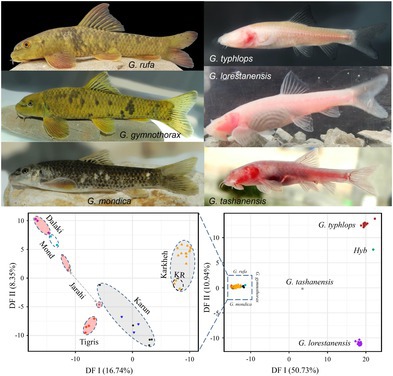

## 1 Introduction

Geographical distributions of biodiversity are influenced by factors including tectonic, climatic, ecological, and human‐mediated events (Hewitt 2000; Mandrak and Crossman 1992; Olafsson et al. 2014; Patsiou et al. 2021; Ruzzante et al. 2020; Toews and Brelsford 2012). Climatic oscillations during the Pleistocene glacial and interglacial periods had and still have a significant impact on the distribution of species, especially primary freshwater fishes, which are often isolated in biogeographic island‐like river catchments (Curry 2007; Mandrak and Crossman 1992; Olafsson et al. 2014; Thomas et al. 2009). However, river and lake ecosystems may have had historic connections, especially due to tectonic activities and glacial cycles with strong changes in precipitation patterns. Furthermore, glacial cycles included the formation of huge continental glaciers (Hughes et al. 2013), resulting in massive sea‐level changes as huge amounts of water became fixed as ice (Spötl et al. 2021). During such events, rivers which today are isolated from each other by shallow seas might have been connected to adjacent rivers, allowing the exchange of biodiversity (Bănărescu 1990). That process is especially important for shallow marine regions, such as the Adriatic Sea, the Sunda Shelf, and the Persian Gulf (Bănărescu 1990; Fagan 2014; Solihuddin 2014).

Here we study biodiversity elements of the Persian Gulf, which not only was dry during glacial maximum phases, but also is situated in a complicated, tectonically active region; dynamics that contributed to the connectivity of the basin's rivers over time (Konyuhov and Maleki 2006). During the Pleistocene/Holocene, the Persian Gulf was repeatedly dried and filled by marine waters during glacial and interglacial periods, respectively (Berends et al. 2021; Dumitru et al. 2021; Hosseinyar et al. 2021; Lambeck 1996; Wood et al. 2012). This situation has been best studied for the late Pleistocene period, when the Persian Gulf or parts of it dried at times of low sea levels, an event expected to have happened during all Pleistocene cold spells, while there were higher sea levels during interglacial periods. During the late Pleistocene, about a dozen such glacial and interglacial fluctuations occurred, among which the most intense cases occurred during the last 0.6–1.0 MY (Berends et al. 2021; Dumitru et al. 2021). During the last glacial maximum (LGM), approximately 30–19 thousand years ago (KYA), sea levels fell to around 120 m below current levels. This regression produced several land bridges in different parts of the world (Berends et al. 2021; Dumitru et al. 2021; Hewitt 2000; Lambeck et al. 2002; Lokier et al. 2015; Rohling et al. 1998; Wood et al. 2012). As a result of marine regression, shallow seas, including the Persian Gulf, with a maximum depth of no more than approximately 100 m, dried (Fagan 2014; Lambeck 1996; Lokier et al. 2015).

Some authors have hypothesized that during the LGM, the Tigris River reached the Sea of Oman (Anderson et al. 2011; Fagan 2014; Lambeck 1996). In that case, all of the currently separate rivers flowing into the Persian Gulf would have been tributaries of the Tigris (Fagan 2014; Lambeck 1996). For freshwater fishes that cannot tolerate marine and brackish environments, the modern marine environment of the Persian Gulf represents an effective barrier to dispersal. However, in the past, freshwater fishes potentially could have dispersed along the river networks that developed in the bed of the Persian Gulf. Therefore, freshwater fishes provide ideal proxies for inferring scenarios that could explain biogeographic patterns in the history of regional rivers.

In the last few decades, an increasing range of genetic markers, including mitochondrial DNA (mtDNA) and nuclear DNA (nDNA) loci, has been utilized to study phylogeography and infer the effects of glaciations on the distribution of species (Hashemzadeh Segherloo et al. 2017; Hashemzadeh Segherloo, Tabatabaei, et al. 2022; Hewitt 2000; Kazyak et al. 2022; Mandrak and Crossman 1992; Olafsson et al. 2014; Rohling et al. 1998). Geographic patterns of mitochondrial and nuclear DNA variation are highly informative for making inferences such as the locations of glacial refugia of populations that later colonized post‐glacial habitats (Dodson et al. 1995; Hashemzadeh Segherloo, Tabatabaei, et al. 2022; Kazyak et al. 2022; Nesbø et al. 1999; Ruzzante et al. 2020). In particular, the integration of data from mtDNA and nDNA loci can enhance the accuracy of inferences regarding biogeography and historical contacts and interactions of populations and species (Gainsford et al. 2020; Hashemzadeh Segherloo et al. 2021; Toews and Brelsford 2012). Among nDNA‐based approaches, the large amount of data produced for either model or non‐model organisms using next‐generation sequencing (NGS) (Allendorf et al. 2010; Andrews et al. 2016; Catchen et al. 2013; Davey et al. 2011) increases the likelihood of detecting even small traces of past events, such as interspecific hybridization and signatures of historic intra‐ and inter‐species contacts, even when using relatively small sample sizes (Hashemzadeh Segherloo et al. 2021; Hashemzadeh Segherloo, Tabatabaei, et al. 2022; Lecaudey et al. 2018).

In the Persian Gulf basin, 14 species within the cyprinid genus *Garra* have been recorded (Jouladeh‐Roudbar et al. 2020; Zamani‐Faradonbe, Keivany, et al. 2021; Zamani‐Faradonbe, Zhang, and Keivany 2021). These species live in warm freshwater and are found over gravel beds in rivers, streams, and springs. While they do not display long‐distance migratory behavior, they may show small‐scale movements within rivers and streams (see Table S1 for more details). Among the noted species in Persian Gulf drainages, only *G. rufa* is widespread and occurs in sympatry, but only rarely in syntopy, with several other *Garra* species over large parts of its geographical distribution. Based on mtDNA data, Hashemzadeh Segherloo et al. (2017) hypothesized that *G. rufa* colonized regional river drainages during the LGM when the dry bed of the Persian Gulf hosted the confluence of currently isolated river drainages.

The diversity of private haplotypes of surface‐dwelling and subterranean populations of *G. rufa* has become known in recent years (Esmaeili et al. 2016; Geiger et al. 2014; Hamidan et al. 2014; Hashemzadeh Segherloo et al. 2012, 2017, 2018; Malek‐Hosseini et al. 2023; Sayyadzadeh et al. 2015; Zamani‐Faradonbe, Keivany, et al. 2021; Zamani‐Faradonbe, Zhang, and Keivany 2021). Among the published mtDNA haplotypes, two geographically isolated common haplotypes exist: one is found in western Persian Gulf basin rivers, including the Tigris, Euphrates, Karkheh, Zohreh, Karun, and Jarahi rivers. The second common haplotype is found in more eastern Persian Gulf basin rivers, including the Dalaki, Mond, and the inland Lake Maharlu basins (Figure S1). From each of these two common haplotypes, several less‐common haplotypes, private to different rivers, have radiated. The existence of two different haplotype radiations in the western and eastern rivers supports the interpretation of colonization of the Persian Gulf drainages by *G. rufa* during the LGM and their postglacial isolation by transgression of marine water. Among the haplotypes radiated from the eastern common haplotype in the Dalaki, one haplotype is shared between the Dalaki and Karun drainages (haplotypes published in Hashemzadeh Segherloo et al. 2017). Assuming that the LGM colonization event was the first wave of the modern *G. rufa* invasion into the eastern rivers of the Persian Gulf, these different haplotype groups in western and eastern river drainages would have evolved after the post‐LGM isolation of western and eastern populations. Based on this inference, there would be no shared rare haplotypes among the radiations within western and eastern rivers due to the vicariance and divergent evolution of the respective haplotypes. The existence of shared haplotypes between the allopatric eastern and western populations of *G. rufa* would, however, be possible under certain scenarios in which: (a) isolated populations of *G. rufa* existed in both eastern and western rivers before the LGM and evolved a set of different haplotypes that during the LGM retreat of marine waters came into secondary contact and exchanged the noted haplotype(s), or alternatively, (b) no *G. rufa* existed in eastern Persian Gulf rivers before the LGM and that mechanisms other than downstream connections—that is, confluences with the extended Tigris River—caused inter‐drainage fish transfers between eastern and western rivers during the post‐LGM period.

In the context of the first scenario, if allopatric populations of *G. rufa* existed in the eastern drainages of the Persian Gulf before the LGM, their genome should show signatures of a population structure caused by their pre‐LGM reproductive isolation. In this case, we should expect the signatures of the secondary contact of these differentiated populations as reciprocal introgression in hybrid zones or within all of the studied river drainages, since all of the pre‐existing populations of *G. rufa* from east and west should, logically, have similar colonization potential from east to west and *vice versa*.

In the context of the second scenario, if *G. rufa* first colonized the eastern rivers only during the LGM retreat of marine water and then fragmented into allopatric eastern and western populations by the transgression of marine water, there would be no signature of reciprocal introgression. Thus, we would expect the haplotype groups and genomic population structure to have evolved in geographic isolation. In this case, mechanisms including river or stream capture would be responsible for the secondary contact(s) between western and eastern Persian Gulf populations of *G. rufa* and any sharing of haplotypes and genotypes between them, since after the post‐LGM rise of the sea to its current level, no downstream connections of eastern river drainages to western rivers would be possible. The effects of river capture events are not expected to be similar to those due to river connections, since river/stream capture events would be accompanied by unidirectional transfers of tributaries, their biota, and population genetic variants from one drainage to other adjacent drainages (Fan et al. 2018).

Hence, our goals in this study were as follows (a) to test the above‐mentioned hypotheses about colonization and secondary contacts of *G. rufa* populations in northeastern and western Persian Gulf river drainages, and (b) to assess possible interactions between *G. rufa* and a number of its sympatric surface‐dwelling and subterranean congeners in the northern Persian Gulf drainages. To do so, we produced single nucleotide polymorphism (SNP) data for 5155 genome‐wide loci and compared genetic markers for several species of *Garra* in the Persian Gulf basin. We included the surface‐dwelling species *G. rufa* , *G. gymnothorax*, and *G. mondica* and the subterranean species *G. typhlops* , *G. lorestanensis*, and *G. tashanensis*.

## 2 Materials and Methods

### 2.1 Sampling

**FIGURE 1:**
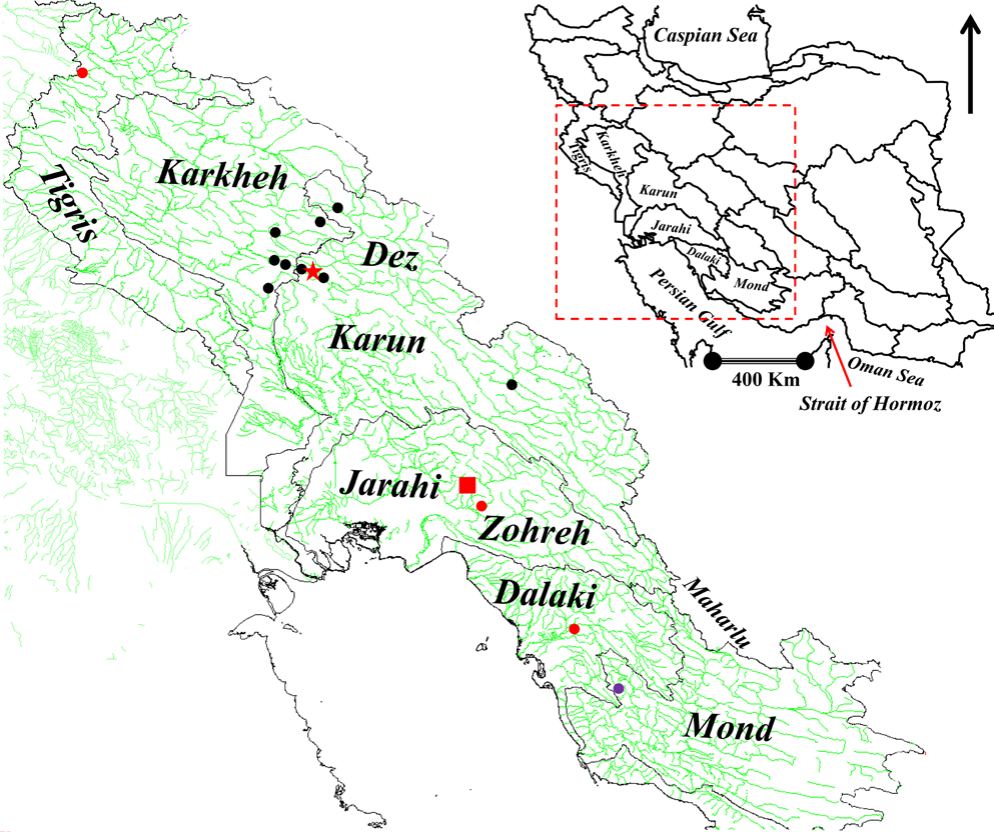
Map showing the studied regions in Iran and sampling sites in the respective river drainages. Species collected at each site are denoted by color of the shapes: Red‐filled circles, *G. rufa* ; Black‐filled circles, *G. gymnothorax*; Red star, *G. typhlops* and *G. lorestanensis*; Red square, *G. tashanensis* and subterranean *G. rufa* ; Purple‐filled circle, *G. mondica*.

To test hypotheses about mechanisms explaining the distribution of *G. rufa* in the northern Persian Gulf drainages and to assess any introgressive hybridization effects of *G. rufa* upon different subterranean and surface‐dwelling species of *Garra*, a total of 26 individuals belonging to *Garra rufa* and *G. gymnothorax* were collected with a dip‐net or via electrofishing using a backpack electrofisher (SAMUS, Bialystok, Poland) (see Figure 1 and Table 1 for metadata). Fish were euthanized by an overdose of clove powder solution. After death, one pectoral fin was clipped and preserved in 95% ethanol. Preserved specimens were kept at −20°C to avoid DNA degradation.

**TABLE 1:** Details of Sampling and data used in this study.

Species	*N* ^a^	Drainage	Sub‐drainge	Tributary	Coordinates		Source of data
					Long	Lat	
*G. gymnothorax*	3	Tigris	Karun	Dopolan	50.6038	31.9157	Hashemzadeh Segherloo et al. (2018)
*G. gymnothorax*	3	Tigris	Karun	Sirum	48.5155	33.0935	This study
*G. gymnothorax*	1	Tigris	Karun	Sirum	48.6329	33.0656	Hashemzadeh Segherloo et al. (2018)
*G. gymnothorax*	1	Tigris	Karun	Sezar	48.9028	33.6448	
*G. gymnothorax*	2	Tigris	Karkheh	Reshno	48.24373	33.1965	This study
*G. gymnothorax*	2	Tigris	Karkheh	Posht Ju	48.30632	33.18091	
*G. gymnothorax*	3	Tigris	Karkheh	Kool‐rast	48.15494	32.91615	
*G. gymnothorax*	4	Tigris	Karkheh	—	48.18631	33.45733	
*G. gymnothorax*	5	Tigris	Karkheh	Hooreh	48.77309	33.51916	
*G. lorestanensis*	14	Tigris	Karun	Dez_Loven Cave	48.5927	33.0773	Hashemzadeh Segherloo et al. (2018)
*G. mondica*	2	Mond	Mond	—	51.73677	28.7958	
*G. rufa*	4	Tigris	Sirvan	—	46.18914	35.11023	
*G. rufa*	3	Dalaki	Dalaki	—	51.26004	29.39704	This study
*G. rufa*	4	Jarahi	Jarahi	Marun	50.30317	30.67037	
*G. tashanensis*	2	Jarahi	Jarahi	Marun_Tashan Cave	50.1754	30.86535	Hashemzadeh Segherloo et al. (2023)
*G. typhlops*	12	Tigris	Karun	Dez_Loven Cave	48.5927	33.0773	Hashemzadeh Segherloo et al. (2018)

^a^ *N*: sample size, coordinates are in decimal degrees.

### 2.2 DNA Extraction

DNA was extracted using the salt extraction method from Aljanabi and Martinez (1997) with an additional treatment with RNase. The quality of the extracted DNA was inspected via electrophoresis through a 1% agarose gel. DNA samples were quantified with a NanoDrop 2000 spectrophotometer (Thermo Scientific), and then DNA concentration was determined using a Picogreen kit (Invitrogen). The concentration of DNA samples was normalized to around 20 ng/μL to standardize sequencing depth among all individuals.

### 2.3 Mitochondrial DNA

The mitochondrial cytochrome oxidase subunit I (*COI*) region was amplified using the primers *FishCOIf*: 5′‐AAYCAYAAAGAYGGYACCCT‐3′ and *FishCOIr*: 5′‐CNGGRTGNCCRAAGAAYCA‐3′ (Lara et al. 2010), to provide mtDNA data for comparison with genomic data to identify any cases of the mito‐nuclear discordance. Further details on PCR conditions can be found in Lara et al. (2010). The amplified DNA fragments were checked via electrophoresis through a 1.5% agarose gel. Sanger sequencing of *COI* amplicons was performed using forward primers on an ABI Prism 3130 sequencer (Applied Biosystems Inc.) at the IBIS sequencing platform (Laval University, Quebec City, Canada; http://www.ibis.ulaval.ca).

### 2.4 Nuclear DNA

The libraries for genotyping‐by‐sequencing (GBS) were prepared following Mascher et al. (2013). Genomic DNA was treated with the *Pst*I and *Msp*I restriction enzymes. The digested DNA samples were then barcoded using individual‐specific oligonucleotide sequences and ligated to adaptors for amplification. Specimens were multiplexed and amplified in a single tube for genotyping on a total of three DNA sequencing chips. Sequencing was performed using Ion Torrent technology at the IBIS sequencing platform.

### 2.5 Data Preparation and Analysis

#### 2.5.1 Mitochondrial DNA Data

Mitochondrial DNA sequences were checked and edited using BioEdit v. 7.2.5 (Hall et al. 2011) and aligned using ClustalW implemented in MEGA7 (Kumar et al. 2016). To find sequences of closely related species or conspecifics (Table 2), a BLAST search (Johnson et al. 2008) was performed (https://blast.ncbi.nlm.nih.gov/Blast.cgi). To visualize mutational relationships among different haplotypes at the intra‐specific and inter‐specific levels, to infer ancestral haplotypes, and to observe geographic structure of haplotypes, a TCS haplotype network (Clement et al. 2002) was constructed using PopART‐1.7 (http://popart.otago.ac.nz).

**TABLE 2:** *COI* sequences from GenBank.

Species	Accession no.	References
*G. rufa*	KM214702.1	Behrens‐Chapuis et al. (2015)
*G. gymnothorax*	KM214735.1	
*G. rufa*	KM214739.1	
*Garra* sp.	KM214741.1	
*Garra* sp.	KM214762.1	
*Garra* sp.	KM214780.1	
*Garra* sp.	KM214782.1	
*G. rufa*	KU722792.1	
*G. rufa*	MN342596.1–MN342597.1	Collins et al. (2012)
*G. rufa*	KX570875.1	Esmaeili et al. (2016)
*G. rufa*	KX570877.1	
*G. rufa*	KM214692.1–KM214695.1	Hamidan et al. (2014)
*G. rufa*	KM214698.1	
*G. rufa*	KM214700.1	
*G. rufa*	KM214712.1	
*G. rufa*	KM214725.1	
*G. rufa*	KM214739.1	
*G. rufa*	KM214743.1	
*G. rufa*	KM214751.1	
*G. rufa*	KM214754.1	
*G. rufa*	KM214761.1	
*G. rufa*	KM214764.1	
*G. rufa*	KM214766.1	
*G. rufa*	KM214772.1	
*G. rufa*	KM214773.1	
*G. rufa*	KM214784.1	
*G. rufa*	KM214792.1	
*G. rufa*	KM214793.1	
*G. rufa*	KM214802.1	
*G. rufa*	KM214805.1	
*G. rufa*	KM214809.1	
*G. rufa*	KU722796.1	
*G. rufa*	JF416296.1 and JF416297.1	Hashemzadeh Segherloo et al. (2012)
*G*. sp.	KM214691.1	Hashemzadeh Segherloo et al. (2017)
*G. rufa*	KM214709.1	
*G. rufa*	KM214711.1	
*G. rufa*	KM214714.1	
*G. rufa*	KM214733.1	
*G. rufa*	KM214760.1	
*Garra* sp.	KM214791.1	
*G. persica*	KM214807.1	
*G. rufa*	KM214810.1	
*G. rufa*	KM373229.1	
*G. rufa*	KM373230.1	
*G. rufa*	KM373231.1	
*G. rufa*	KM373232.1	
*G. rufa*	KM373233.1	
*G. rufa*	MT900746.1–MT900747.1	Kirchner et al. (2021)
*G. rufa*	OR573692.1–OR573697.1	Malek‐Hosseini et al. (2023)
*G. tashanensis*	OR574022.1–OR574032.1	
*G. rufa*	KT808681.1	Sayyadzadeh et al. (2015)
*G. rufa*	MN254980.1–MN254981.1	Unpublished
*G. rufa*	MN255070.1–MN255106.1	Unpublished
*G. rufa*	KU568825.1–KU568826.1	Van der Walt et al. (2017)
*G. rufa*	NC_022941.1	Yang et al. (2012)
*G. rufa*	MN254982.1–MN255069.1 and MT159908.1	Zamani‐Faradonbe, Keivany, et al. (2021)

#### 2.5.2 Genomic Data

To prepare a unified data set, we integrated the raw genome reads produced via Genotyping‐By‐Sequencing reported in Hashemzadeh Segherloo et al. (2018, 2023) with the raw genomic data produced in this study. Data preparation, genotyping, and filtration were done using STACKS v2.62 (Catchen et al. 2013; https://catchenlab.life.illinois.edu/stacks/) and stacks_workflow v2.62 (https://github.com/enormandeau/stacks_workflow). Briefly, cutadapt v1.18 (‐e 0.2 ‐m 50) was used to trim reads for quality. Samples were demultiplexed with process_radtags v2.62 (‐c ‐r ‐t 80 ‐q ‐s 0 ‐‐barcode_dist_1 2 ‐E phred33 ‐‐renz_1 pstI ‐‐renz_2 mspI). We then ran ustacks (‐m 4 ‐M 3 ‐N 5 ‐H ‐‐deleverage), cstacks (‐n 1), sstacks, tsv2bam, gstacks, and population (‐p 2 ‐r 0.6 ‐‐fasta‐loci ‐‐vcf) to produce a minimally filtered VCF file containing the genotypes of the samples. After these STACKS steps, we proceeded to filter the SNPs as follows. We considered all the samples as part of the same group and filtered the SNPs (05_filter_vcf_fast.py, params: 3 90 0 3) so that all the genotypes had a minimum coverage of 3; we kept only those SNPs for which the sample had at most 10% missing data and for which at least 3 samples had the rare allele. Using a modified *HDplot* approach (McKinney et al. 2017), we then sought to remove SNPs that displayed signs of paralogy or over‐merging (scripts 08, 09 and 10 from stacks_workflow). Keeping only canonical SNPs, referred to as singletons, we removed SNPs that were in high linkage disequilibrium with script 11_extract_unlinked_snps.py. To measure linkage between a pair of SNPs, only samples that possessed the rare allele for either of the SNPs and had no missing data in both SNPs were retained. Using these samples, SNPs were deemed in linkage when genotypes were the same for both SNPs more than 50% of the time within one STACKS locus. In these cases, we kept only the left‐most SNP from the locus.

Using ADMIXTURE v1.3.0 (Alexander et al. 2009), the number of different samples was explored for *K* values from 1 to 10. Based on the cross‐validation (*CV*) error values returned by ADMIXTURE, and by comparing the admixture graphs, a value of *K* = 4 was retained and used for the purpose of missing genotype imputation. For each SNP, missing genotypes were generated by drawing two random alleles. The frequencies of alleles 1 and 2 for the random draw were determined by using the frequencies of the different groups identified by admixture and the relative membership of the given sample to these groups.

##### 2.5.2.1 Population Clustering

To clarify genomic clusters of *Garra* species and cases of introgression among the studied drainages, admixture analysis and discriminant analysis of the principal components (DAPC) were performed. Admixture analysis was run with 200 bootstrap replicates for population groups (*K*) of 1–10 using ADMIXTURE v1.3.0. The best‐supported number of genetic clusters was selected based on a 10‐fold cross‐validation error (*CV*), for which the lowest *CV* value denotes the most possible number of clusters. For DAPC, we grouped populations based on sampling locality and performed the analysis using the Adegenet 2.0.0 R package (Jombart and Collins 2015). To visualize the clustering pattern among the closely related species including *G. rufa* , *G. gymnothorax*, and *G. mondica*, a separate DAPC analysis was performed on genomic data of only these species.

##### 2.5.2.2 Inference of Past Gene Flow

To infer past hybridization/introgression events, we reconstructed a neighbor‐net using SNP data with SplitsTree V. 4.14.6 (Huson and Bryant 2005) and calculated *D*‐statistics for all possible trios of populations/species using Dsuite (Malinsky et al. 2021). As we aimed to test introgression in a biogeographic context, populations of each species and also different species were identified using a combination of the scientific names and the river drainages which they inhabit. In the *D‐*statistics (ABBA‐BABA) test of introgression, the frequencies of different patterns of shared derived alleles are compared among three ingroups (P_1_, P_2_, and P_3_) and an outgroup (P_0_; *G. typhlops* here). We selected *G. typhlops* as the outgroup because it does not show any signature of introgression in its own admixture cluster or in admixture clusters of other species analyzed here. Under the null hypothesis of no gene flow and only the effects of incomplete lineage sorting (ILS), the two discordant genealogies (ABBA and BABA) are expected to occur at equal frequencies, since ILS is a random process and would not cause a considerable frequency change in favor of one pattern over the other. Therefore, a significant excess of one pattern over the other (|*Z*| > 3) is considered a signature of introgression between a pair of ingroup species/populations. In this case, a BBAA pattern is considered as the tree shape in the absence of introgression or hybridization. Dsuite also calculates the *f*_4_‐ratio, which quantifies the proportion of admixture in each population/species triplet. Further, to identify signatures of introgression among the internal branches of the species tree, the output of Dsuite along with the rooted species tree were fed into the Fbranch module of Dsuite. The *f*‐branch (*f_b*) statistic applies evidence from the *f*_4_‐ratio to specific internal branches that enable detection of introgression between branches of the tree. All Dsuite commands were run using default parameter settings.

##### 2.5.2.3 Species Tree Reconstruction

To infer phylogenetic relationships and the geographic origin of each population/species among the studied species of *Garra*, while avoiding the effects of incomplete lineage sorting, a species tree under a multispecies coalescent model was reconstructed using the SVDQuartets method implemented in PAUP* v.4.0 (Swofford 2003). In this case, we assumed that populations or species which nest within a shared clade or cluster are of the same geographic origin or affected by introgression from other members of the corresponding clade (see Hashemzadeh Segherloo, Tabatabaei, et al. 2022). To reconstruct the species tree, 100,000 quartets were evaluated, and in cases where the number of quartets was less, all possible quartets were evaluated. A hundred bootstrap replicates were performed to assess branch support. The trees were selected using Quartet Fiduccia and Mattheyses (QFM) quartet assembly. To avoid the interpretation of IUPAC ambiguity codes as missing data, the “distribute” option under “handling of ambiguities” section of PAUP* was selected.

## 3 Results

### 3.1 Mitochondrial Data

Here, we focus upon *G. rufa* and analysis of the mitochondrial *COI* data from different populations that have already been published (*n* = 197, Esmaeili et al. 2016; Geiger et al. 2014; Hamidan et al. 2014; Hashemzadeh Segherloo et al. 2012, 2017, 2018; Hashemzadeh Segherloo, Najafi Chaloshtory, et al. 2022; Malek‐Hosseini et al. 2023; Sayyadzadeh et al. 2015; Zamani‐Faradonbe, Keivany, et al. 2021; Zamani‐Faradonbe, Zhang, and Keivany 2021) and from *G. rufa COI* sequences newly produced in this study (*n* = 4). Based upon the haplotype network reconstructed for a 596‐bp *COI* sequence from published and new data (Figure S1), *G. rufa* populations shared two common and 28 less‐common haplotypes with 1–4 bp differences from their respective common haplotype—by common haplotypes, we mean the haplotypes which exist in two or more river drainages. In the Jarahi drainage, one haplotype showing a 4‐bp difference from the common haplotype was from a subterranean population of *G. rufa* . The second common haplotype plus seven haplotypes with 1–3 bp differences were exhibited by fish from the isolated Maharlu Lake basin and the Dalaki and Mond river drainages. One of the less‐common haplotypes from the Dalaki River drainage was also found in the Karun River drainage.

### 3.2 Single Nucleotide Polymorphisms (SNPs)

A total of 5155 SNPs were exhibited by the populations/species under study. Two individuals of *G. tashanensis* had a maximum of approximately 30% missing data. Of the remaining 63 individuals, only eight had more than 10% missing data. These eight individuals included four from the Karun (*G. gymnothorax*: 10.8%–13.2% missing data), one from the Tigris ( *G. rufa* : 11.7% missing data), two from the Mond (*G. mondica*: 10.8%), and one from the Loven (*G. lorestanensis*: 11.4% missing data) river drainages (Figure S2).

#### 3.2.1 Population Clusters

**FIGURE 2:**
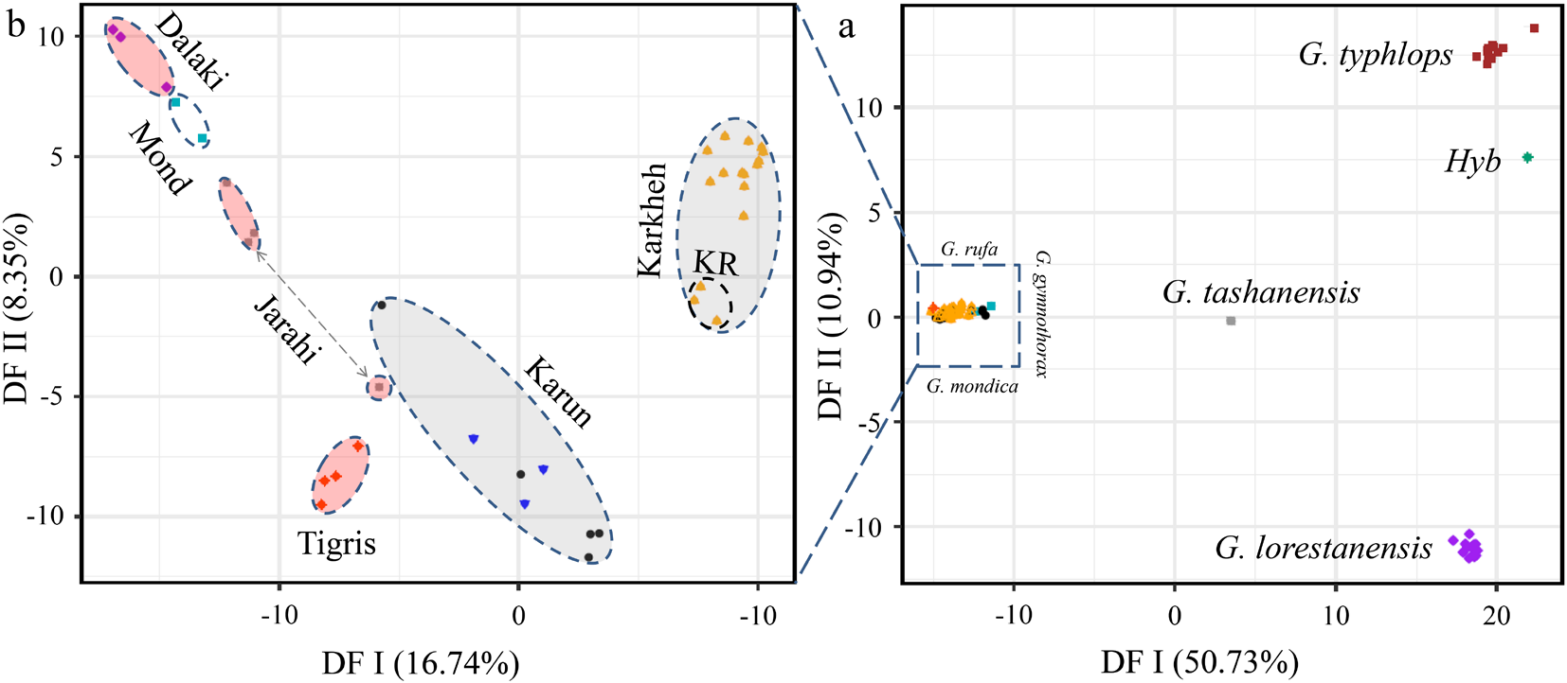
Clustering patterns of *Garra* spp. along the first two discriminant functions (DF I and II) for: (a) all species analyzed, and (b) *G. rufa* , *G. gymnothorax*, and *G. mondica*. The dashed ovals in panel (b) are used only to identify specimens belonging to each separate river. In panel (b), the black circles and inverted blue triangles encircled as the Karun cluster correspond to specimens from the Dez and Karun rivers, respectively. Hyb, a hybrid individual between *G. typhlops* and *G. lorestanensis*; KR, Kool‐rast Stream. The dashed arrow denotes specimens from the Jarhi River.

Results of discriminant analysis of principal components (DAPC) (Figure 2a) showed four clearly separate clusters, including: (a) *G. typhlops* , (b) *G. lorestanensis*, (c) *G. tashanensis*, and (d) *G. rufa* , *G. gymnothorax*, and *G. mondica* along the first two discriminant axes (50.73% and 10.94% of the total genetic variation, respectively). All populations/species belonging to *G. rufa* , *G. gymnothorax*, and *G. mondica* were grouped in a single DAPC cluster along the first and second discriminant axes.

To visualize the finer clustering pattern among the latter group of species, a second DAPC analysis was performed using SNP data belonging only to *G. rufa* , *G. gymnothorax*, and *G. mondica*. Six clusters were identified when only these three species were included. Along the first axis, which accounted for 16.74% of the genetic variation, *G. gymnothorax* from the Karun drainage was located intermediate between *G. rufa* from the Tigris and *G. gymnothorax* from the Karkheh drainages (Figure 2b). *Garra rufa* from the Dalaki and Jarahi drainages were genetically similar to *G. mondica* of the Mond drainage, but *G. rufa* from the Tigris showed a clear genetic distinction from *G. rufa* of the Dalaki and Jarahi drainages. In the Karkheh drainage, the cluster of *G. gymnothorax* from the Kool‐rast stream was genetically distinct from other *G. gymnothorax* of the Karkheh drainage along the second axis (8.35% of genetic variation; Figure 2b).

**FIGURE 3:**
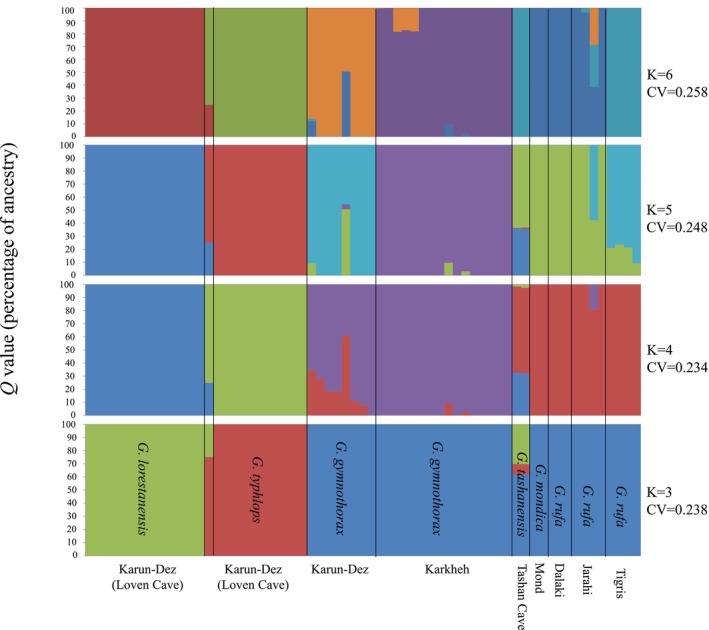
Admixture graphs for different numbers of clusters (*K* = 3–6). Horizontal axis (*X*‐axis) denotes individuals from different drainages and vertical axis (*Y*‐axis) shows the percentage of different genomic clusters within each individual. The scientific name corresponding to members of each cluster is indicated on admixture graph for *K* = 3. Black vertical lines delineate different species or conspecific individuals from different localities.

The admixture analysis on both unimputed and imputed genotype data resulted in the same admixture clustering patterns. Based on the cross‐validation error (*CV*) values 3, 4, and 5, population clusters (*K*) were all good candidates. For *K* = 4, which had the lowest cross‐validation error (*CV*), distinct clusters were found for: (a) *G. typhlops* , (b) *G. lorestanensis*, (c) *G. rufa* from the Tigris, Jarahi, and Dalaki, and *G. mondica* from the Mond drainages, and (d) *G. gymnothorax* from Karkheh and Karun drainages (Figure 3). For *K* = 4, seven of eight *G. gymnothorax* individuals from the Karun drainage and both *G. tashanensis* individuals from Tashan Cave in the Jarahi drainage were identified as hybrids.

#### 3.2.2 Past Gene Flow

**FIGURE 4:**
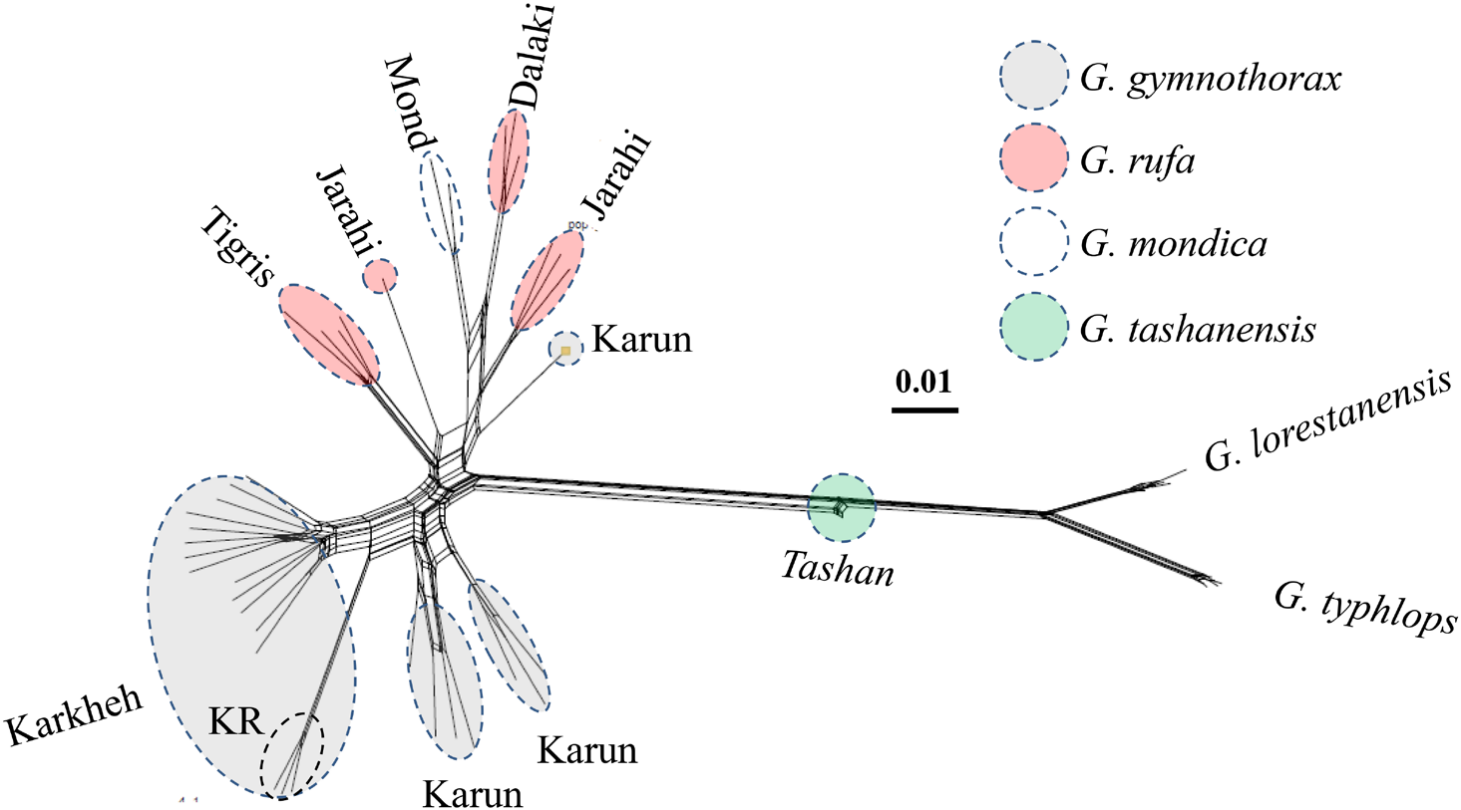
Neighbor network reconstructed using SNP data. As indicated in legend, each color denotes a species, and the river or locality of origin for each cluster is indicated beside the dashed ovals or circles. KR denotes Kool‐rast Stream (a seasonal tributary of the Karkheh River). As the Dez river is a tributary of the Karun River, we have only used Karun for those specimens.

**FIGURE 5:**
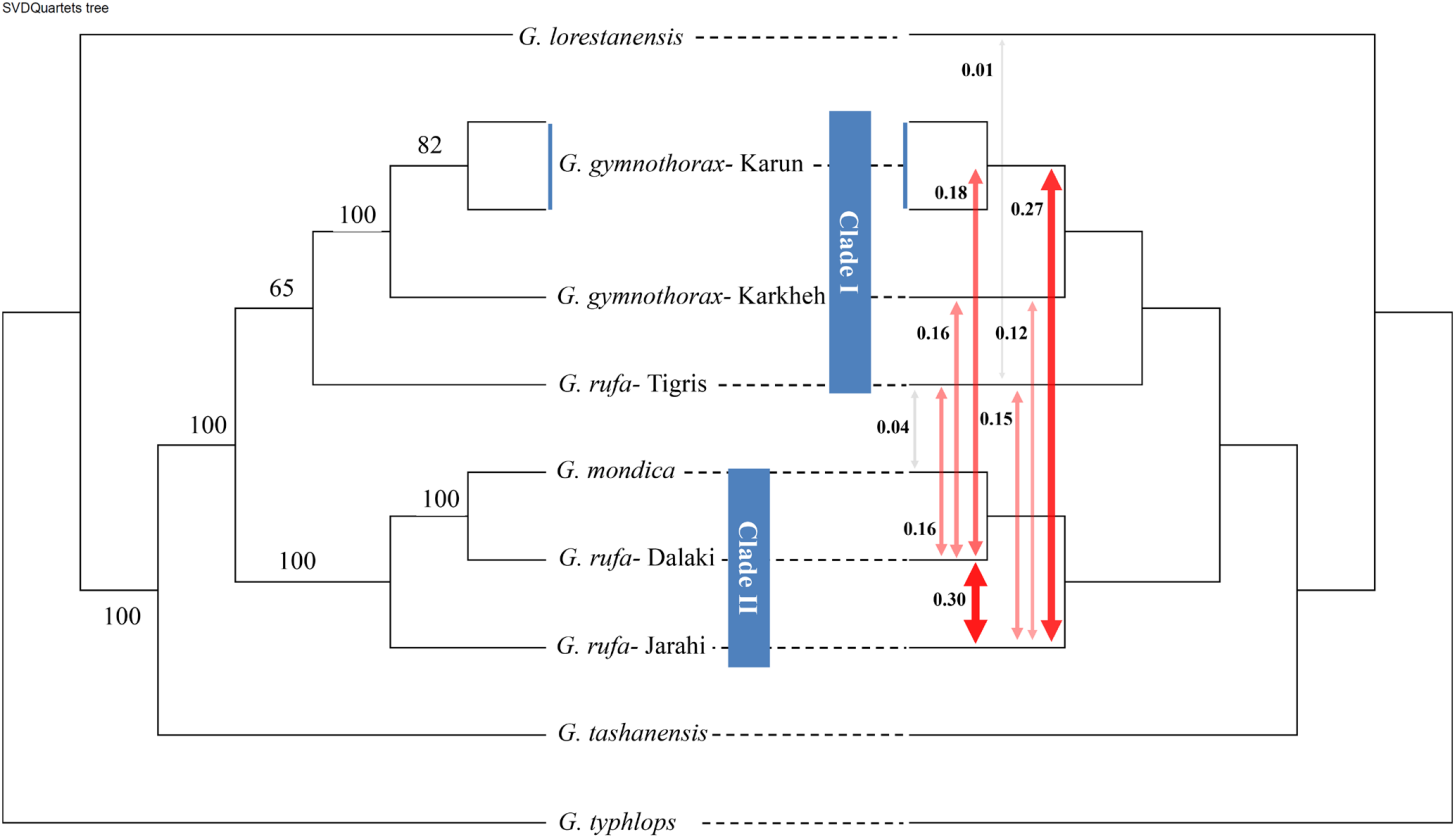
SVDQuartets species tree reconstructed using SNP data in PAUP*. Numbers along branches are bootstrap support values (left panel). On the right panel, geneflow between different internal branches of the species tree is illustrated based on the rates estimated using the Fbranch module implemented in Dsuite. The arrows and the values beside them denote introgression/geneflow and f‐branch (*f‐b*) statistic. The color and thickness of arrows show the strength of geneflow—light colors and thinner arrows indicate weaker geneflow, and darker colors and thicker arrows indicate stronger geneflow signals.

In the Neighbor‐net (Figure 4), each population/species formed its own cluster. *Garra tashanensis* was intermediate to the *G. typhlos/G. lorestanensis* cluster and the clusters belonging to other *Garra* species considered here. *Garra gymnothorax* from the Karun drainage appeared to be introgressed. The *D‐*statistics detected highly significant (|*Z*| > 3; *p* < 0.01) introgression/hybridization between: (a) *G. rufa* (P_2_; the Dalaki River) and *G. gymnothorax* (P_3_; the Karun River; *f*_4_‐ratio = 18%), (b) *G. rufa* (P_2_; the Dalaki River) and *G. gymnothorax* (P_3_; the Karkheh River; *f*_4_‐ratio = 16%), (c) *G. rufa* (P_2_; the Jarahi River) and *G. gymnothorax* (P_3_; the Karun River; *f*_4_‐ratio = 19%), and (d) *G. rufa* (P_2_; the Dalaki River) and *G. rufa* (P_3_; the Tigris River; *f*_4_‐ratio = 16%) (Table 3). Further, weaker (3 > |*Z*| > 2; 0.01 < *p* < 0.05) signatures of introgression/hybridization were detected between: (a) *G. mondica* (P_2_; the Mond River) and *G. rufa* (P_3_; the Jarahi River; *f*_4_‐ratio = 12%), (b) *G. rufa* (P_2_; the Jarahi River) and *G. rufa* (P_3_; the Tigris River; *f*_4_‐ratio = 10%), and (c) *G. rufa* (P_2_; the Tigris River) and *G. mondica* (P_3_; the Mond River; *f*_4_‐ratio = 6%) (Table 3). The Fbranch test (Figure 5) that integrates the species tree with the *D*‐statistics detected geneflow between *G. rufa* of the Dalaki River and *G. rufa* of the Jarahi (*F*‐statistic = 0.30) and the Tigris (*F*‐statistic = 0.17) rivers; between *G. gymnothorax* of Karun (*F*‐statistic = 0.18) and Karkheh (*F*‐statistic = 0.16) rivers; and between *G. rufa* from the Jarahi and *G. gymnothorax* of the Karun (*F*‐statistic = 0.27) and Karkheh (*F*‐statistic = 0.12) rivers, as well as *G. rufa* of the Tigris (*F*‐statistic = 0.13–0.16). This test also detected weak gene flow between *G. rufa* of the Tigris and *G. mondica* (*F*‐statistic = 0.04), *G. rufa* of the Dalaki (*F*‐statistic = 0.08) rivers, and *G. lorestanensis* (*F*‐statistic = 0.01).

**TABLE 3:** Results of Dsuite ABBA‐BABA tests (Patterson's *D*) to assess geneflow among the *Garra* spp. considered in this study using a four‐population/species framework with *G. typhlops* as outgroup. Only tests with *Z‐*scores ≥ 2 and *p* ≤ 0.05 are reported.

P_1_	P_2_	P_3_	*D*‐statistic	*Z*‐score	*p*	*f*_4_‐ratio (%)	BBAA	ABBA	BABA
*G. mondica*	*G. rufa* (Dalaki)	*G. gymnothorax* (Karun)	0.14	4.22	< 0.01	18	150.16	82.16	62.29
*G. mondica*	*G. rufa* (Dalaki)	*G. gymnothorax* (Karkheh)	0.14	4.12	< 0.01	16	145.88	80.64	60.95
*G. rufa* (Dalaki)	*G. rufa* (Jarahi)	*G. gymnothorax* (Karun)	0.1	3.52	< 0.01	19	144.71	103.88	84.96
*G. mondica*	*G. rufa* (Dalaki)	*G. rufa* (Tigris)	0.16	3.41	< 0.01	16	136.49	87.38	63.08
*G. gymnothorax* (Karkheh)	*G. rufa* (Tigris)	*G. lorestanensis*	0.18	2.93	< 0.01	3	464.86	17.54	12.06
*G. gymnothorax* (Karkheh)	*G. tashanensis*	*G. lorestanensis*	0.24	2.34	< 0.01	8	172.17	28.53	17.46
*G. gymnothorax* (Karkheh)	*G. mondica*	*G. rufa* (Jarahi)	0.06	2.13	< 0.05	12	69.69	113.20	99.79
*G. rufa* (Dalaki)	*G. rufa* (Jarahi)	*G. rufa* (Tigris)	0.08	2.12	< 0.05	10	128.14	103.18	88.29
*G. gymnothorax* (Karun)	*G. rufa* (Tigris)	*G. lorestanensis*	0.15	2.11	< 0.05	2	467.36	16.75	12.41
*G. rufa* (Jarahi)	*G. rufa* (Tigris)	*G. lorestanensis*	0.14	2.03	< 0.05	2	482.71	16.89	12.63
*G. gymnothorax* (Karkheh)	*G. rufa* (Tigris)	*G. mondica*	0.07	2.02	< 0.05	6	107.66	84.55	73.21

#### 3.2.3 Species Tree

In the species tree using *G. typhlops* as out‐group—selected based upon its admixture cluster and position in the DAPC results—several species, including *G. tashanensis*, *G. mondica*, *G. rufa* , and *G. gymnothorax*, were found to nest within one cluster with 100% support (Figure 5; bootstrap support, *BS* = 100). Two clades/sub‐clades can be distinguished within this cluster: (a) ‘clade I’ (not strongly supported, *BS* = 65) in which *G. gymnothorax* from the Karkheh and Karun and *G. rufa* from the Tigris drainages nested, and (b) ‘clade II' (strongly supported, *BS* = 100), which contained *G. rufa* from the Dalaki and Jarahi drainages and *G. mondica*. *Garra tashanensis* was found to be the sister species to both clades.

## 4 Discussion

This study analyses mtDNA and genome‐wide SNP data for six surface‐dwelling and subterranean *Garra* species from Persian Gulf drainages to infer natural historical biogeographical processes of isolation and secondary contact. Our findings provide inferences on processes that shaped the present‐day distributions and genetic structure of the considered *Garra* species in the region. We focus upon the relatively broad distribution of *G. rufa* populations and their interactions with other, more geographically confined *Garra* species. We develop scenarios explaining the colonization of different river drainages by *G. rufa* and provide a geographic hypothesis to justify our inferences regarding colonization events. Finally, we briefly discuss the effect of *G. rufa* colonization and hybridization upon surface‐dwelling and subterranean *Garra* species in the studied region.

### 4.1 On the Invasion of *Garra rufa*

Based on our genomic data, there are two major clusters in surface‐dwelling *Garra* spp.: one comprising populations/species in the Mond and Dalaki (eastern) drainages and the second comprising populations/species in western (Karun, Karkheh, and Tigris) drainages. This general pattern is in agreement with a colonization event followed by isolation—most probably colonization of eastern drainages during the LGM‐related sea‐level retreat, followed by isolation mediated by Holocene rise of marine water into the Persian Gulf (Hashemzadeh Segherloo et al. 2017; Teimori et al. 2012, 2015; Fagan 2014; Lambeck 1996). Along with this general genetic structure, which accords with the overwhelming pattern observed among mtDNA haplotypes, genomic SNP data show signatures of introgression from *G. rufa* of the Dalaki drainage into *G. rufa* of the Jarahi drainage. However, *G. rufa* in the Jarahi drainage has western mtDNA haplotypes, but its position on the species tree or in the population distribution graphs produced using genome‐wide SNP data is pulled toward the population clusters of *G. rufa* from the adjacent Dalaki drainage and nests within the eastern clade on the species tree, which is contradictory to post‐glacial isolation of Dalaki and Mond from the western rivers including the Tigris, Karkheh, Karun, and Jarahi river drainages. Concordant with this pattern in the species tree, highly significant signatures of introgression between the *G. rufa* of the Dalaki and Jarahi rivers are detected in the *D*‐statistic test of introgression (|*Z*| = 3.52, *p* < 0.01, *f‐b* = 0.30). We infer this mismatch or mito‐nuclear discordance and the significant signal of introgression between *G. rufa* of the Jarahi and Dalaki drainages as the signature of a westward transfer of *G. rufa* from the Dalaki drainage. Naturally, sink populations or species receiving immigrants should show various extents of genotypic similarity to the source population/species from which individuals emigrated, that is, more geneflow leads to more genotypic resemblance of the sink (Jarahi) to the source (Dalaki) population. On the other hand, no highly significant signatures of admixture from the western *G. rufa* genome or mtDNA—published here and in other studies—could be found in the Dalaki or Mond drainage populations—only a weak signature of introgression between *G. rufa* of the Tigris drainage and *G. mondica*; |*Z*| = 2.02, *p* < 0.05, *f‐b* = 0.04 was detected; hence, we suggest that these cases of unidirectional admixture were not related to fish transfers via downstream river catchment expansion events during the LGM. The latter case could have provided the opportunity for bidirectional movement of eastern and western *G. rufa* , for which no evidence could be provided by our analyses. Instead, we hypothesize that secondary contact between eastern and western populations of *G. rufa* was limited to unidirectional transfer(s) of fish from east to west during the post‐LGM period. In addition, the common haplotype of the eastern *G. rufa* exists in fish from both the Lake Maharlu and the Mond drainages which are adjacent to the Dalaki drainage. Lake Maharlu, an isolated basin which does not have any outlet to the Persian Gulf. Hence, we propose that unidirectional movement of eastern *G. rufa* into the Jarahi drainage and Lake Maharlu basin is explained by river capture event(s) after post‐LGM flooding of marine water into the Persian Gulf. As a result of river capture, a tributary or a part of the catchment of a river is transferred to a neighboring river system due to tectonic movements, sedimentation, or erosion (Fan et al. 2018; Bishop 1995; Walker et al. 2011; Willett et al. 2014). The simple fold of the Zagros Mountains where the headwaters of the Zohreh River (a western river adjacent to the Jarahi), Dalaki, and Mond rivers and the Maharlu Lake basin are closely located is a geologically active region with reported cases of river catchment rearrangements (Walker et al. 2011). As the Zohreh River is geographically adjacent to the Jarahi River, and no strong geomorphological barrier exists between these two rivers on the Khuzestan Plain, migration of fish from the Zohreh to the Jarahi or even to the Karun drainage is possible via flooding events. The highly significant signatures of introgression between *G. rufa* of the Dalaki and Jarahi river drainages and *G. gymnothorax* of the Karun and Karkheh river drainages reinforce the possibility of such movements (|*Z*| > 3.52, *p* < 0.01). However, more clarification of the mechanism(s) responsible for the unidirectional movement of the eastern *G. rufa* to the Zohreh and Jarahi drainages awaits follow‐up studies with a more complete sampling scheme to include fish from different rivers and different *Garra* species.

### 4.2 Possible Effects of *G. rufa* Dispersal Upon Other *Garra* Species

Among the other *Garra* species considered here, *G. mondica* from the Mond drainage is highly divergent from *G. rufa* in its mtDNA, and this is why it had been described as a distinct species (Sayyadzadeh et al. 2015). However, its genomic cluster was identical to *G. rufa* from the Dalaki drainage in DAPC, Admixture, and species tree analyses. *Garra mondica* has mtDNA clearly distinct from *G. rufa* , but it is not being supported as an independent nDNA group by our nuclear data. Furthermore, morphological characters provided by Sayyadzadeh et al. (2015) and Zamani‐Faradonbe and Keivany (2021) are highly divergent and we were not able to find consistent morphological differences between *G. mondica* and *G. rufa* based on our own materials studied (Freyhof et al. 2025). This situation only allows one conclusion: the specimens with *G. mondica* mtDNA must be identified as *G. rufa* . It needs to be confirmed in the future if this will be the case for all individuals of *G. mondica* from its type locality. We suspect that there had been an endemic *Garra* species in the Mond River drainage in the past. But this species vanished following the invasion of *G. rufa*, and only the mitochondria of the extinct species remain in a few populations of *G. rufa* . Overall, according to our genomic data and the morphological data (Freyhof et al. 2025), “*G. mondica*” is an extinct species, from which only the mitochondria survived in the cells of *G. rufa* as a remnant of past introgressive hybridization.

*Garra gymnothorax* in the Karun and Karkheh drainages is a second species with a mtDNA highly diverged from *G. rufa* , without known morphological differences to *G. rufa* (Zamani‐Faradonbe and Keivany 2021; Freyhof et al. 2025). *Garra gymnothorax* shows significant signatures of introgression from *G. rufa* of the Dalaki and Jarahi river drainages (|*Z*| > 3.52, *p* < 0.01), which can be related to the reduced bootstrap support of their respective clade with *G. rufa* of the Tigris River in the species tree. However, the close relationship of *G. gymnothorax* to *G. rufa* in the species tree indicates that they have also experienced hybridization with *G. rufa* of the Tigris drainage, which is in agreement with its morphological similarity with *G. rufa* (Zamani‐Faradonbe and Keivany 2021; Freyhof et al. 2025). This hybridization is biogeographically plausible since the Karkheh and Karun rivers drain to the downstream reaches of the Tigris drainage. Based on the patterns revealed in the population clusters (both DAPC and Admixture graphs), compared to *G. gymnothorax* of the Karkheh River, *G. gymnothorax* from the Karun River drainage are more oriented toward or affected by *G. rufa* of the Tigris, while both these rivers are connected to the Tigris River. The explanation may lie in the ecological and population or demographic differences between the Karkheh and Karun drainages. For example, in populations with small effective population sizes, introgressed alleles can reach fixation more quickly than in large populations, or the ecological conditions in the Karun may have been more favorable for *G. rufa* .

*Garra tashanensis* is a cave‐dwelling species highly diverged in its mitochondrial DNA from all other *Garra* species considered here (Hashemzadeh Segherloo, Najafi Chaloshtory, et al. 2022; Mousavi‐Sabet et al. 2016), but in the species tree reconstructed here with *G. typhlops* as the outgroup, this species nested in a monophyletic clade with *G. rufa* , *G. gymnothorax*, and *G. mondica* (*BS* = 100), which we think can be due to the introgressive effects of *G. rufa* ; otherwise, *G. tashanensis* should not nest in a clade with the *G. rufa* complex with absolute bootstrap support. In this regard, our data show signatures of allele‐sharing between *G. lorestanensis* and *G. tashanensis*—based on the admixture graph for *K* = 5 and Patterson's *D*‐statistics (|*Z*| > 2.34, *p* < 0.01)—and also between *G. rufa* and *G. tashanensis*—there were no significant *D*‐statistics, but in the Admixture test, signatures of hybridization were detected. For the case of *G. lorestanensis* and *G. tashanensis* it is challenging to accept that these species have introgressed, since *G. lorestanensis* is a subterranean species inhabiting aquifers of the Karun River drainage and *G. tashanensis* is another subterranean fish species native to aquifers of the Jarahi River drainage (Hashemzadeh Segherloo et al. 2025; Mousavi‐Sabet et al. 2016). There may be other reasons for the allele‐sharing between these two species, for example, convergent selective pressures in subterranean habitats leading to the convergent evolution or formation of their genotypes. We cannot explain more about this observation at this time, and postpone further inferences to follow‐up studies. Introgression between *G. rufa* and *G. tashanensis* makes more sense, since Malek‐Hosseini et al. (2023) reported a stygobiotic population of *G. rufa* living in a seasonal spring in syntopy with *G. tashanensis*. Epigean populations of *G. rufa* are widespread in the region and can penetrate into the subterranean habitat, when and where these habitats flow out to streams. This case is an interesting question to be treated in further surveys which will include the troglomorphic *G. rufa* and *G. tashanensis*.

## 5 Conclusions

Overall, we present original data and inferences supporting the case for post‐LGM secondary contact between eastern and western *G. rufa* populations that became isolated by post‐Pleistocene Sea level rise of the Persian Gulf. We suggest river capture as a probable mechanism explaining secondary dispersal of *Garra* lineages from the Dalaki to the Zohreh and Jarahi drainages. Further, a high mito‐nuclear discordance was detected in *G. mondica* and *G. gymnothorax*, which is inferred to be the consequence of introgressive hybridization with *G. rufa* . Overall, the high genomic similarities and overlapping morphological features among *G. rufa* , *G. gymnothorax*, and *G. mondica* is more compatible with a scenario in which these groups are considered as hybrid *G. rufa* carrying mtDNA lineage of the swamped species, rather than separate species. Further studies with a more complete taxon sampling and geographic coverages integrating genomic (nDNA) and mtDNA data along with detailed morphological analyses can provide better understanding of the biogeographic patterns and taxonomy of different *Garra* lineages in the studied region.

## Author Contributions

**Iraj Hashemzadeh Segherloo:** conceptualization (lead), data curation (lead), formal analysis (lead), funding acquisition (lead), resources (equal), writing – original draft (lead), writing – review and editing (equal). **Murtada D. Naser:** formal analysis (equal), resources (equal), writing – review and editing (equal). **Amaal G. Yasser:** formal analysis (equal), resources (equal), writing – review and editing (equal). **Seyedeh Narjes Tabatabaei:** formal analysis (equal), investigation (equal), methodology (equal), writing – original draft (equal), writing – review and editing (equal). **Fardin Shaloei:** formal analysis (equal), resources (equal), writing – review and editing (equal). **Shirin Rahmati:** investigation (equal), resources (equal), writing – original draft (equal). **Hojat Keshani:** investigation (equal), resources (equal), writing – original draft (equal). **Aghil Mansouri:** investigation (equal), resources (equal), writing – original draft (equal). **Eric Normandeau:** data curation (equal), formal analysis (equal), writing – original draft (equal), writing – review and editing (equal). **Jörg Freyhof:** conceptualization (equal), writing – original draft (equal), writing – review and editing (equal). **Eric Hallerman:** formal analysis (equal), writing – review and editing (lead). **Erik García‐Machado:** formal analysis (equal), writing – review and editing (equal). **Alieh Changizi:** investigation (equal), resources (equal), writing – original draft (equal). **Louis Bernatchez:** conceptualization (equal), funding acquisition (lead), resources (lead).

## Funding

This work is supported by a NSERC (Canada) Discovery grant (http://www.nserc‐crsng.gc.ca) to Louis Bernatchez, grant number 688MIGRD94 to Iraj Hashemzadeh Segherloo by Shahr‐e‐Kord University (www.sku.ac.ir), a short‐term scholarship (V3 program) from the Fonds de Recherche Québécois sur la Nature et les Technologies (FRQNT: www.frqnt.gouv.qc.ca), and a research fellowship awarded by the Alexander von Humboldt Foundation, Germany (https://www.humboldt‐foundation.de) to Iraj Hashemzadeh Segherloo.

## Conflicts of Interest

The authors declare no conflicts of interest.

## Supporting information


**Figure S1:** TCS haplotype network reconstructed for a 596‐bp COI sequence of *Garra rufa* using Popart‐1.7.
**Figure S2:** Proportion of missing data in the studied individuals.
**Table S1:** Distribution and ecology of *Garra* species considered in this study.

## Data Availability

The VCF file used for the analyses is available as a Supporting Information to the publication. The *COI* sequences produced in this study can be accessed from GenBank using accession numbers: PX061849–PX061852.
