# Supplementary material for: New Insights Into the Biogeography of Six Garra Species (Teleostei: Cyprinidae) in the Persian Gulf Basin
Source: Ecol Evol. 2026 Apr 27;16(4):e73463. doi: 10.1002/ece3.73463 (PMC13112081; doi:10.1002/ece3.73463)
Supplement: Supplementary file 1 — Figure S1: TCS haplotype network reconstructed for a 596‐bp COI sequence of Garra rufa using Popart‐1.7. Figure S2: Proportion of missing data in the studied individuals. Table S1: Distribution and ecology of Garra species considered in this study. [file ECE3-16-e73463-s001.zip › 3_Supplementary Figure 1 TCS haplotype network reconstructed for a 596.docx]

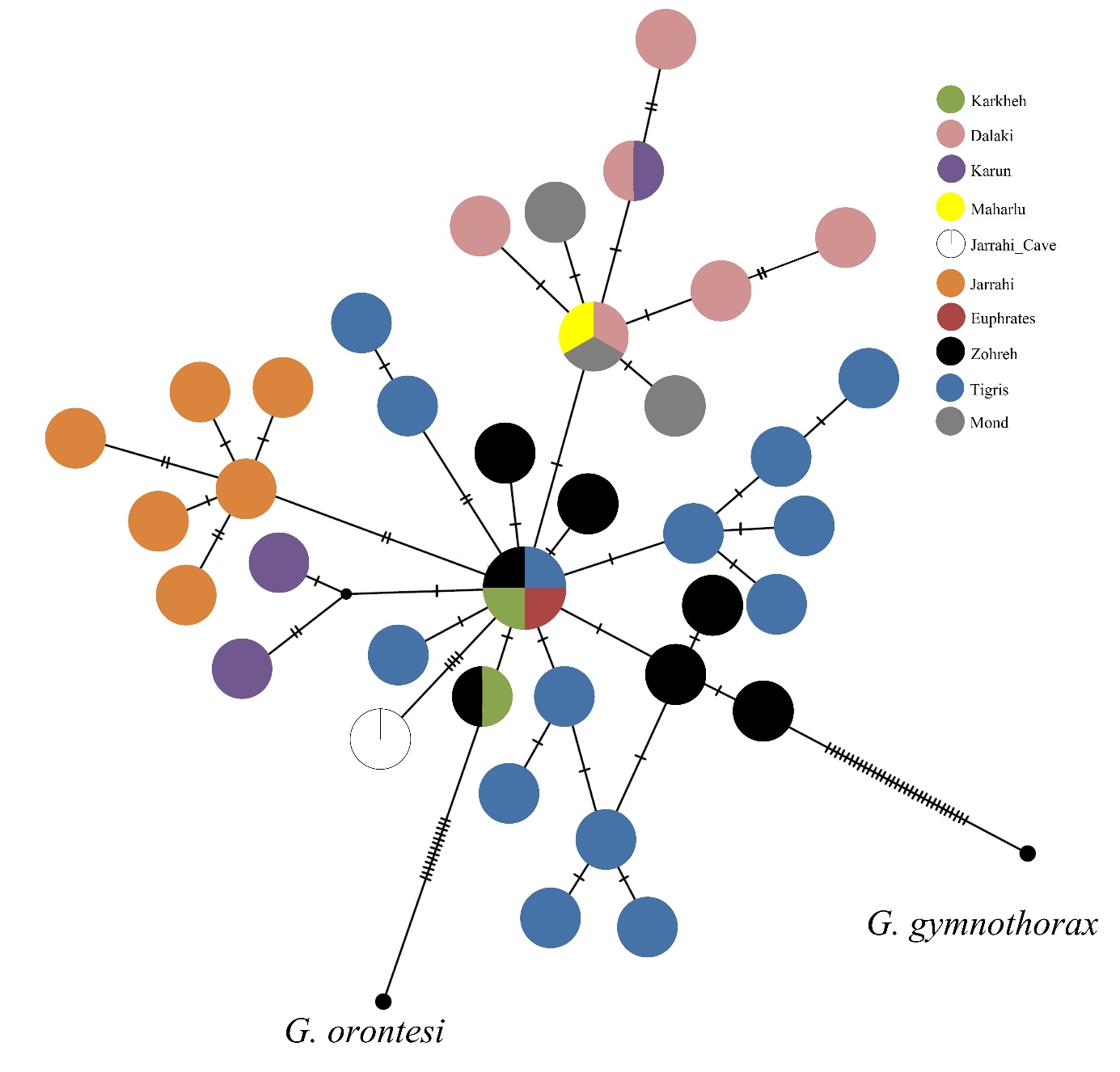


**Supplementary Figure 1** TCS haplotype network reconstructed for a 596-bp *COI* sequence of *Garra rufa* using PopART-1.7. Each color corresponds to a river drainage as denoted in the legend. The hatch marks along connecting lines denote mutational differences between each pair of haplotypes. Black unnamed points are hypothetical haplotypes which lead to the observed haplotypes. *Garra gymnothorax* and *G. orontesi* are included in the haplotype network to root the network and also to determine ancestral haplotypes.
