# Supplementary material for: New Insights Into the Biogeography of Six Garra Species (Teleostei: Cyprinidae) in the Persian Gulf Basin
Source: Ecol Evol. 2026 Apr 27;16(4):e73463. doi: 10.1002/ece3.73463 (PMC13112081; doi:10.1002/ece3.73463)
Supplement: Supplementary file 1 — Figure S1: TCS haplotype network reconstructed for a 596‐bp COI sequence of Garra rufa using Popart‐1.7. Figure S2: Proportion of missing data in the studied individuals. Table S1: Distribution and ecology of Garra species considered in this study. [file ECE3-16-e73463-s001.zip › 3_Supplementary Figure 2 Proportion of missing data in the studied individuals.docx]

**Supplementary Figure 2** Proportion of missing data in the studied individuals. Abbreviations: *G. mon*, *G. mondica*; *G. g, G. gymnothorax*; hyb, hybrid between *G. typhlops* and *G. lorestanensis*; *G. r*, *G. rufa*; *G. tash*, *G, tashanensis*.
