# Supplementary material for: New Insights Into the Biogeography of Six Garra Species (Teleostei: Cyprinidae) in the Persian Gulf Basin
Source: Ecol Evol. 2026 Apr 27;16(4):e73463. doi: 10.1002/ece3.73463 (PMC13112081; doi:10.1002/ece3.73463)
Supplement: Supplementary file 1 — Figure S1: TCS haplotype network reconstructed for a 596‐bp COI sequence of Garra rufa using Popart‐1.7. Figure S2: Proportion of missing data in the studied individuals. Table S1: Distribution and ecology of Garra species considered in this study. [file ECE3-16-e73463-s001.zip › 3_Supplementary Table 1.docx]

Supplementary Table 1 Distribution and ecology of *Garra* species considered in this study.

| Species | Distribution | Migratory | Feeding | Habitat | References |
| --- | --- | --- | --- | --- | --- |
| *G. rufa* | Euphrates, Tigris, Karkheh, Karun, Jarahi, Zohreh, Dalaki, Mond river drainages | No, but can migrate in freshwater courses | Phytoplankton, Protozoa, and Nematoda | Warmwater rivers, streams, springs, and subterranean habitats | Hashemzadeh Segherloo et al. (2017), Malek-Hosseini et al. (2023), Demirci et al. (2016) |
| *G. gymnothorax* | Karkheh and Karun river drainages | No, but can migrate in freshwater courses |  | Warmwater streams, rivers and springs | Hashemzadeh Segherloo et al. (2017) |
| *G. typhlops* | Dez and Karun river drainages | Not known | Omnivore, feeding on Planktons | Subterranean | Hashemzadeh Segherloo et al. (2012, 2018, 2025), Farashi et al. (2014) |
| *G. lorestanensis* |  |  |  |  |  |
| *G. tashanensis* | Jarahi River Drainage | Not known | No detailed data, but most probably omnivore, feeding on Planktons | Subterranean | Mousavi-Sabet et al. (2016)  Farashi et al. (2014) |
| *G. mondica* | Mond River Drainage | No, but can migrate in freshwater courses | No data, but most probably Phytoplankton, Protozoa, and Nematoda | Small springs in the upper Mond River drainage | Sayyadzadeh et al. (2015)  Demirci et al. (2016) |

**References**

Demirci, S., S. Y. Ozdilek, and E. Simsek. 2016. “Study on Nutrition Characteristics of *Garra rufa* on the River Asi.” *Fresenius Environmental Bulletin* 25, no. 12: 5999–6004.

Farashi, A., M. Kaboli, H. R. Rezaei, M. R. Naghavi, and H. Rahimian. 2014. “Plankton Composition and Environmental Parameters in the Habitat of the Iranian Cave Barb (*Iranocypris typhlops*) in Iran.” Animal Biodiversity and Conservation 37, no. 1: 13–21.
